# Type 2C Protein Phosphatases *MoPtc5* and *MoPtc7* Are Crucial for Multiple Stress Tolerance, Conidiogenesis and Pathogenesis of *Magnaporthe oryzae*

**DOI:** 10.3390/jof9010001

**Published:** 2022-12-20

**Authors:** Jules Biregeya, Wilfred M. Anjago, Shu Pan, Ruina Zhang, Zifeng Yang, Meilian Chen, Abah Felix, Huxiao Xu, Yaqi Lin, Oswald Nkurikiyimfura, Yakubu Saddeeq Abubakar, Zonghua Wang, Wei Tang

**Affiliations:** 1 State Key Laboratory of Ecological Pest Control for Fujian and Taiwan Crops, Fujian Agriculture and Forestry University, Fuzhou 350002, China; 2 Fujian Universities Key Laboratory for Plant-Microbe Interaction, College of Life Science, Fujian Agriculture and Forestry University, Fuzhou 350002, China; 3 Fuzhou Institute of Oceanography, Minjiang University, Fuzhou 350108, China; 4 Department of Biochemistry, Ahmadu Bello University, Zaria 810103, Nigeria

**Keywords:** blast fungus, protein phosphatases, phosphorylation, pathogenicity

## Abstract

Protein kinases and phosphatases catalyze the phosphorylation and dephosphorylation of their protein substrates, respectively, and these are important mechanisms in cellular signal transduction. The rice blast fungus *Magnaporthe oryzae* possesses 6 protein phosphatases of type 2C class, including MoPtc1, 2, 5, 6, 7 and 8. However, only very little is known about the roles of these phosphatases in filamentous fungi. Here in, we deployed genetics and molecular biology techniques to identify, characterize and establish the roles of MoPtc5 and MoPtc7 in *M. oryzae* development and pathogenicity. We found that during pathogen-host interaction, *MoPTC7* is differentially expressed. Double deletion of *MoPTC7* and *MoPTC5* suppressed the fungal vegetative growth, altered its cell wall integrity and reduced its virulence. The two genes were found indispensable for stress tolerance in the phytopathogen. We also demonstrated that disruption of any of the two genes highly affected appressorium turgor generation and Mps1 and Osm1 phosphorylation levels. Lastly, we demonstrated that both MoPtc5 and MoPtc7 are localized to mitochondria of different cellular compartments in the blast fungus. Taken together, our study revealed synergistic coordination of *M. oryzae* development and pathogenesis by the type 2C protein phosphatases.

## 1. Introduction

*Magnaporthe oryzae* is a filamentous fungus that causes the blast disease of rice which results in severe losses of global rice production. Due to the economic significance and experimental tractability of *M. oryzae*, it is considered a perfect pathosystem for studying host-pathogen interactions [1]. Infection wise, *M. oryzae* is similar to other important pathogens of cereals [2,3]. Hence there is possibility of establishing at least a broad-spectrum drug that targets common disease determinants in the fungi for effective disease management. The rice blast fungus causes up to 15% of the potential worldwide rice harvest, and this is adequate food for more than sixty million people [4]. *M. oryzae* is highly destructive to rice partly because of its ability to adopt different cycles (polycyclic pathogen) within a growing season [5]. *M. oryzae* infects rice plants at all stages of development and attacks the leaves, stems, nodes and panicles [2]. For the infection cycle, conidia are dispersed by wind onto the host leaf surfaces where each conidium develops a germ tube, the tip of which swells into an infection structure called appressorium [6]. Mature appressorium then accumulates turgor pressure (up to about 8 MPa) that mechanically ruptures the leaf cuticle to enable the pathogen enter the plant cells [7]. Under favorable environmental conditions, lesions occur within 3–4 days of infection, after which new conidia are produced to start a new disease cycle [3]. Previously, some studies revealed that a group of signaling pathways regulate the pathogenicity of *M. oryzae*, including MAPK and cAMP pathways which affect conidiation as well as appressorium formation [8,9]. 

Cellular processes such as cell growth and differentiation, pH regulation, cell cycle, morphogenesis and apoptosis are largely regulated via phosphorylation and dephosphorylation of proteins by kinase and phosphatase groups of enzymes [10]. Protein phosphorylation constitutes a very essential mechanism by which higher plants and animals regulate cellular processes [11]. In particular, signal transductions are transmitted through phosphorylation and dephosphorylation of some enzymes and regulatory proteins. Protein phosphatases of serine/threonine family are classified into PPM and PPP superfamilies. Members of the latter superfamily have both regulatory and catalytic subunits [12], while members of the former are monomeric enzymes [13].Generally, both groups are share common inhibitors; and PP2C phosphatase requires Mg^2+^ or Mn^2+^ as coenzymes [14]. 

Within the PP2C family, Ptc1 is identified in *Saccharomyces cerevisiae* to be involved in the regulation of MAPK pathways [15]. In *S. cerevisiae*, Ptc1 regulates stress response pathways [15,16]. Furthermore, Ptc1 was shown to mediate tRNA splicing, organelles distribution, inheritance, cation homeostasis and sporulation in budding yeast [17]. In *S. cerevisiae*, Ptc2 proteins share 60% identity, and both proteins are structurally different from Ptc1 as each of them possesses a 170 residues extension at its carboxyl terminus, which allows them achieve maximum activity [16]. Protein phosphatases play vital roles in preserving cell viability when the cells are exposed to agents that compromise DNA integrity, thereby maintaining genome stability [18]. in *S. cerevisiae*, cell cycle is partly regulated by synergistic effects of Ptc2 and Ptc3 on dephosphorylation of Thr-169 in Cdc28 kinase [19]. Ptc4 is localized in the cytoplasm in *S. cerevisae* and its overexpression significantly reduced the phosphorylation activity of Hog1 [20]. Ptc2 and Ptc4 participate in cell wall integrity, and possibly limit the hyperphosphorylation of CWI pathway [21]. 

There is limited information on the functions of Ptc5 and Ptc7 in filamentous fungi like *M. oryzae*, but in budding yeast, these proteins have been shown to regulate pyruvate dehydrogenase activity by dephosphorylating Pda1, an E1 α-subunit of the PDH complex in mitochondria [22]. Ptc7 regulates the PDH activity by dephosphorylating the canonical citrate synthase of the tricarboxylic acid cycle CIT1, thereby maintaining its proper dimerization during its activity. Deletion of *AP2C1* and *PP2C5* generated plant mutants with abnormal physiologic and phenotypic characteristics [23]. More so, both Ptc5 and Ptc7 were previously demonstrated to be involved in rapamycin-induced stress resistance [24]. Therefore, understanding the relationship between these proteins (Ptc5 and Ptc7) and the pathogenesis and development of *M. oryzae* would be critical in handling the menace of the rice blast infection, thereby strengthening the global food security. As such, we employed gene deletion approach to investigate the functions of MoPtc5 and MoPtc7 in relation to the pathogenesis and development of *M. oryzae*. Our findings indicated that deletion of *MoPTC5* and *MoPTC7* perturbs the fungal vegetative growth, conidiation, conidiophores development, pathogenicity and Mps1 and Osm1 phosphorylation levels. Our results also reveal that the type 2C protein phosphatases are required for multiple stress tolerance in the rice blast pathogen.

## 2. Materials and Methods

### 2.1. Strains and Culture Conditions

Guy11 strain of *M. oryzae* was used in this study as the WT (wild type), and the various mutant strains were generated from the Guy11 background. Complete media was used to culture the fungal strains [25]. Liquid complete media (CM) were used for the growth of mycelia for nucleic acids extractions and protoplast preparation. TB3 medium (200 g sucrose, 3 g casamino acids, 3 g yeast extract and 7 g agar in 1 L of distilled water) was used for transformation, growth and screening of transformants.

Rice bran media (RBM: 40 g of rice bran in 1 L of ddH_2_O) was used for induction of conidiation and the fungal cultures were incubated in the dark at 28 °C for 10 days, and then exposed to light condition for another 3 days. Conidia were harvested, washed with sterile double distilled water (ddH_2_O), filtered and counted under a light microscope with the help of a hemocytometer. For growth assays, we cultured the strains on complete media (CM: 6 g yeast extract, 6 g casamino acid, 10 g sucrose in 1 L of water), Starch yeast media (SYM: 10 g starch extract, 2 g yeast extract, 3 g sucrose) and RBM.

### 2.2. Targeted Gene Deletion and Complementation

For *MoPP2C* gene deletion, homologous recombination approach was adopted. The flanking regions of the phosphatase genes were amplified using the appropriate primers (Table S1). The PCR products were then ligated with hygromycin phosphotransferase (hph) gene by overlap PCR. *M. oryzae* protoplasts were prepared, into which the above constructs were transformed following the protocols previously reported [26]. A TB3 medium was used for the screening of transformants in the presence of hygromycin B (Roche Applied Science, Penzberg, Germany). Appropriate mutants were subjected to Southern blot (elaborated in Section 2.5 below) for confirmation. 

For complementation assay, complementation vectors were designed by amplifying full length ORFs (and their respective promoters) of the deleted genes and ligating them with GFP. These products were then cloned into PKNTG plasmid, respectively, after digestion with a mixture of *Kpn*I and *Hind*III restriction enzymes. The recombinant DNA construct was sequenced to ensure successful cloning. The *MoPTC5* and *MoPTC7* constructs were respectively transformed into the protoplasts of Δ*Moptc5* and Δ*Moptc7* mutants and the transformants screened by PCR using the appropriate primers. For confirmation, GFP signals were analyzed by confocal microscopy (Nikon, Tokyo, Japan).

### 2.3. Infection Assays, Cuticle Penetration and Incipient Cytorrhysis Assay

To perform rice infection assay, we prepared conidia suspensions (5 × 10^4^ spores/mL) from Guy11, Δ*Moptc5*, Δ*Moptc7*, Δ*Moptc7*Δ*Moptc5*, Δ*Moptc5_C,* and Δ*Moptc7_C* strains and sprayed them on 3-week-old blast susceptible rice seedling (*Oryza sativa*, CO39). The seedlings were then incubated in the dark at 28 °C for 24 h in a humid growth chamber, and later subjected to 12/12 h of light/dark photoperiod. Leaves were then collected from the seedlings for observation of disease lesions at 7 days post infection (dpi). For barley infection assay, 10 days old barley leaves were subjected to same treatments and incubated under same conditions. Leaves from the infected plants were collected for observation at 7 days. 

To study the cuticle penetration potential, we dropped 10 μL of conidia suspensions (5 × 10^4^ spores/mL) from the various strains onto 10 days old barley leaves, and incubated the leaves at 28 °C for 30 h, 48 h and 60 h under humid condition. A confocal microscope (Nikon, Japan) was then used to check for cuticle penetration and growths of invasive hyphae. 

The turgor pressures in the appressoria of the strains were analyzed by incipient cytorrhysis assay on hydrophobic coverslips after 24 h of treatments with glycerol solutions (1, 2, and 3 M). The number of collapsed appressoria was counted under a light microscope.

### 2.4. Microscopy

While a confocal microscope was used for investigation of subcellular localizations, light microscopy using Olympus DP80 (Tokyo, Japan) was utilized for conidiophores formation assay, cytorryhsis incipient assay, invasive hyphae development and appressorium penetration and glycogen degradation.

### 2.5. Southern Blot Analysis, Extraction of Total RNA, qRT-PCR and Gene Expressions

For southern blot assay, we extracted DNA from the fungal hyphae following cetyl trimethyl ammonium bromide (CTAB) method [25]. Restriction digestion, gel electrophoresis, blotting, probe labeling, ligation and hybridization were conducted using a commercial kit (Roche), following the manufacturer protocol. Briefly, the gels were immersed in alkaline buffer for 15 min with mild shaking to depurinate the fractioned DNA and denature them by soaking in the gel blotting solution (0.4 M NaOH, 0.6 M NaCl) for 30 min. The gels were then moved to neutralization buffer (1.5 M NaCl, 0.5 M Tris -HCl pH 7.5) for 30 min before capillary blotting on hybond-N (GE-healthcare). The gel blots were achieved by putting the inverted gels on the sheet of filter papers. The hybond–N membrane was then placed on the gels and covered with filter papers. The filter papers were being regularly changed after getting wet for 2 days. The membrane was UV cross-linked immediately probed or wrapped for storage in saran wrap. 

To examine the gene transcription profiles in planta at different growth stages, we sprayed Guy11 conidia on 3 weeks old CO-39 rice, the leaf samples were then collected at different time points (4 h, 8 h, 12 h, 24 h, 48 h and 72 h). Next, we extracted total RNA using an RNA extraction kit (Eastep super RNA) and cDNA was prepared following the protocols described in the manual (accurate biology, Hunan, China). qPCR was performed where actin gene was used as an internal control. Mean Ct values were normalized as previously described [27]. 

For analysis of the expressions of conidiation responsive and chitinase encoding genes in the various strains, we inoculated the strains in liquid CM placed in an incubator shaker at 28 °C, 110 rpm for 3 days. After this period, samples were collected and total RNA extracted using a kit (Eastep super RNA). cDNA were prepared following the aforementioned protocols. qPCR was conducted and tubulin gene was used as an internal control. Mean Ct values were normalized as previously described [27]. The primers used are listed in Table S1. 

### 2.6. Bioinformatics Analysis

Protein sequence alignment was performed using DNAMAN software. The protein sequences were obtained from NCBI (National Center for Biotechnology Information) database by BLAST analysis of the protein sequences of PP2C homologs in *S. cerevisiae*. The identified homologs in *M. oryzae* were confirmed by BLASTp search at the fungi and oomycetes genomics resources database (http://fungidb.org/fungidb/, accessed on 10 January 2022). For domain prediction and phylogenetic analysis, amino acid sequences of MoPP2c and their respective orthologs were fed into Pfam based domain prediction analysis software and the various domains identified, while phylogenetic analyses were performed using MEGA x software, adopting Neighbor-Joining method and 1000 bootstrap. 

### 2.7. Cell Wall Integrity, Osmotic and Oxidative Stresses and Cell Wall Thickness Assays

Sodium dodecyl sulphate (SDS, 0.01%), Calcofluor White (CFW, 200 µg/mL) and Congo Red (CR, 200 µg/mL) were supplemented into CM agar, respectively, on which the fungal strains were grown for analysis of cell wall stress sensitivity. For osmotic stress assay, CM media was supplemented with sodium chloride (NaCl, 1 M), potassium chloride (KCl, 1 M) and sorbitol (1 M). For oxidative stress assay, the CM was supplemented with hydrogen peroxides (5 mM and 10 mM H_2_O_2_). The strains were cultured and their colony diameters measured at 10 dpi. Inhibition rates were calculated as previously described [28]. For cell wall thickness assay, we inoculated the strains in liquid CM and incubated under constant agitation for three days. Next, mycelia were harvested and dried. Transmission electron micrographs of the transverse section of the cell walls were taken by scanning electron microscopy (SEM).

### 2.8. Western Blot Assay

The fungal mycelia were used for extraction of total proteins as previously described [29]. The extracted proteins were subjected to SDS-PAGE and fixed on PDVF (polyvinylidene fluoride) membranes, and the proteins of interest were spotted by antiphospho p44/42 and P42/44 primary antibodies. HRP-Rabbit were used as conjugated secondary antibodies. A western blot detection Kit (Advansta, San Jose, CA, USA) was used for signals visualization using an imaging system (Tanon 5200). Pictures were processed using imaging software.

### 2.9. Subcellular Localization Assays 

One Step Cloning Kit (Vazyme, Nanjing, China) was used for the construction of MoPtc5 and MoPtc7-GFP and RFP vectors. The vectors were transformed into the WT background, respectively. The transformants were screened by GFP and RFP fluorescence signal analyses at different developmental stages in planta, by confocal microscopy. Images were processed using NIS-viewer software.

## 3. Results

### 3.1. Phylogenetic Analysis of MoPtc5 and MoPtc7

**Figure 1:**
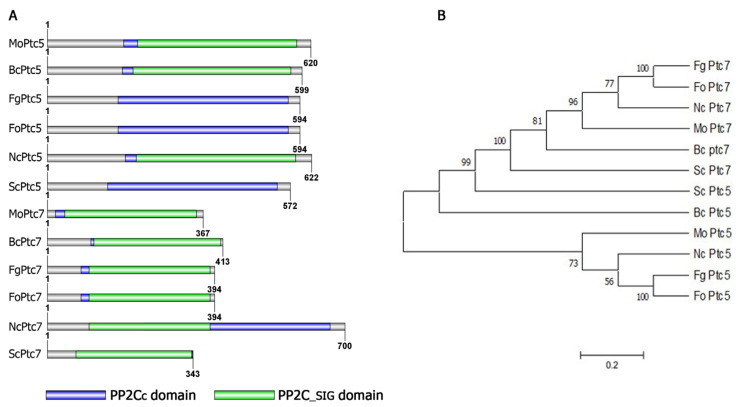
Domain architecture and phylogenetic analysis of Ptc5 and Ptc7 in different fungi. (**A**) Domain architecture of Ptc5 and Ptc7 orthologs in in different organisms. (**B**) A phylogenetic tree generated using MEGA X software and a UPGMA test with 1000 Boot straps replications was conducted. Fg: *Fusarium gamineum*; Fo: *Fusarium oxyporum*; Nc: *Neurospora crassa*; Mo: *Magnaporthe oryzae*; Bc: *Botytis cinera*; and Sc: *Saccharomyces cerevisae*.

The putative homologs of Ptc5 and Ptc7 were identified in *M. oryzae* by BLASTp search at the fungi and oomycetes database using *S. cerevisiae* Ptc5 and Ptc7 sequences as queries. The BLASTp hits identified two copies of *PP2C* genes MGG_03154 and MGG_00166 in the rice blast genome, which were subsequently named *MoPTC5* and *MoPTC7*, respectively. Protein structure and domain prediction analysis showed that MoPtc5 protein contains a single PP2C domain while MoPtc7 has a PP2C and PP2C-SIG domains (Figure 1A). Phylogenetic analysis revealed that MoPtc5 shared a close homology with its orthologs in *Neurospora crassa* and *Fusarium oxyporum* while MoPtc7 has close homology with its orthologs in *Botrytis cinera* and *N. crassa* (Figure 1B).

### 3.2. MoPtc5 and MoPtc7 Are Differentially Expressed during Pathogen-Host Interaction 

**Figure 2:**
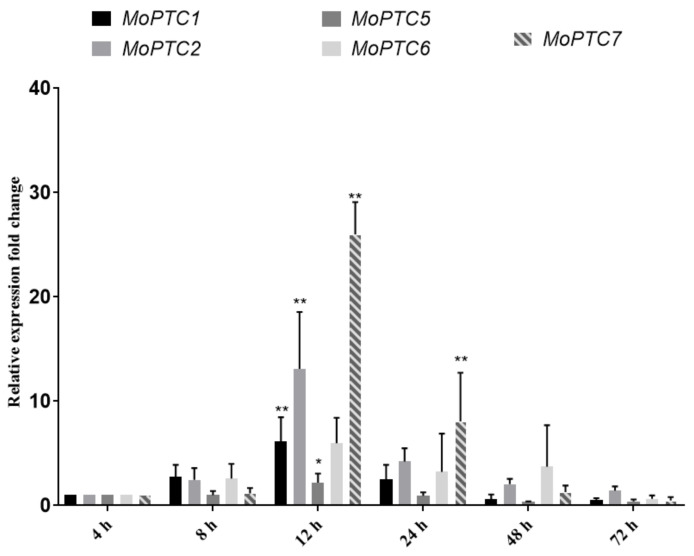
Transcriptional expression patterns of *M. oryzae* type 2C genes during interaction with host. Quantitative realtime (q-PCR) was used to check the genes expressions at the indicated time points. The 4 h time point was taken as background control with tubulin gene serving as internal control. The experiments were repeated three times with three technical replicates each. Error bars represent standard deviations while asterisks show significant differences at different *p*-values (* *p* < 0.1; ** *p* < 0.01). The data were analyzed by one-way ANOVA using Tukey’s multiple-comparison test.

To gain a clue on the importance of MoPtc5 and MoPtc7 in *M. oryzae* virulence, we first decided to check the expression patterns of all the type 2C genes (*MoPTC1*, *MoPTC2*, *MoPTC5*, *MoPTC6* and *MoPTC7*) in Guy11 during host infection, at different time points. To achieve this, we harvested conidia from the WT (Guy11) strain and sprayed them on 21-day-old rice seedlings. We then quantified the type 2C genes expression levels at 8, 12, 24, 48 and 72 h. We found that, with the exception of *MoPTC6*, all the type 2C genes were significantly expressed at 12 hpi (hours post inoculation), while only *MoPTC7* was significantly expressed at 24 hpi (Figure 2). These results show that MoPtc5 and MoPtc7 could be involved in the development and virulence of *M. oryzae*. 

### 3.3. MoPtc5 and MoPtc7 Redundantly Contribute to Vegetative Growth of M. oryzae

**Figure 3:**
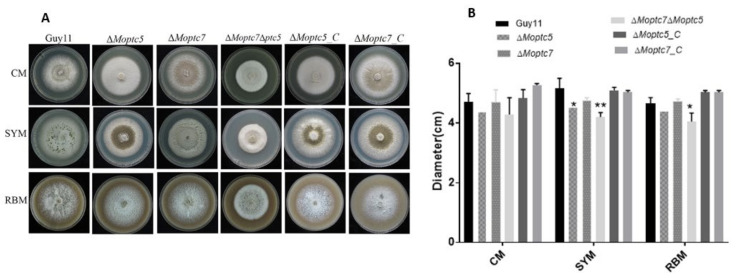
Synergistic roles of MoPtc5 and MoPtc7 in *M. oryzae* vegetative growth of. (**A**) The growths of the various strains on different media plates. CM, SYM and RBM were used for the growth of the fungal mycelia and incubated in the dark for 10 days at 28 °C. (**B**) Bar graphs showing the colony diameters of the various strains on the indicated media. Asterisks show significant differences at different *p*-values (* *p* < 0.1; ** *p* < 0.01) while error bars represent standard deviations from three independent replicates. The data were analyzed by one-way ANOVA using Tukey’s multiple-comparison test.

In order to examine the roles played by MoPtc5 and MoPtc7 in the vegetative growth of the fungal pathogen, we generated Δ*Moptc5*, Δ*Moptc7* and Δ*Moptc7*Δ*Moptc5* mutants by homologous recombination approach (Figure S1). The mutants (plus the controls) were then cultured on CM, SYM and RBM and incubated for 10 days at 28 °C. Compared to the controls, the colony diameters of Δ*Moptc5* and Δ*Moptc7* on CM and RBM were not significantly different, while Δ*Moptc5* revealed significant difference on SYM (Figure 3A,B). In contrast, a significant reduction was observed in the growth of the double deletion mutant Δ*Moptc7*Δ*Moptc5* on both SYM and RBM media. These suggest the redundant roles of MoPtc5 and MoPtc7 in the vegetative growth of the blast fungus. 

### 3.4. MoPtc5 Is Involved in Asexual Reproduction of the Rice Blast Fungus

**Figure 4:**
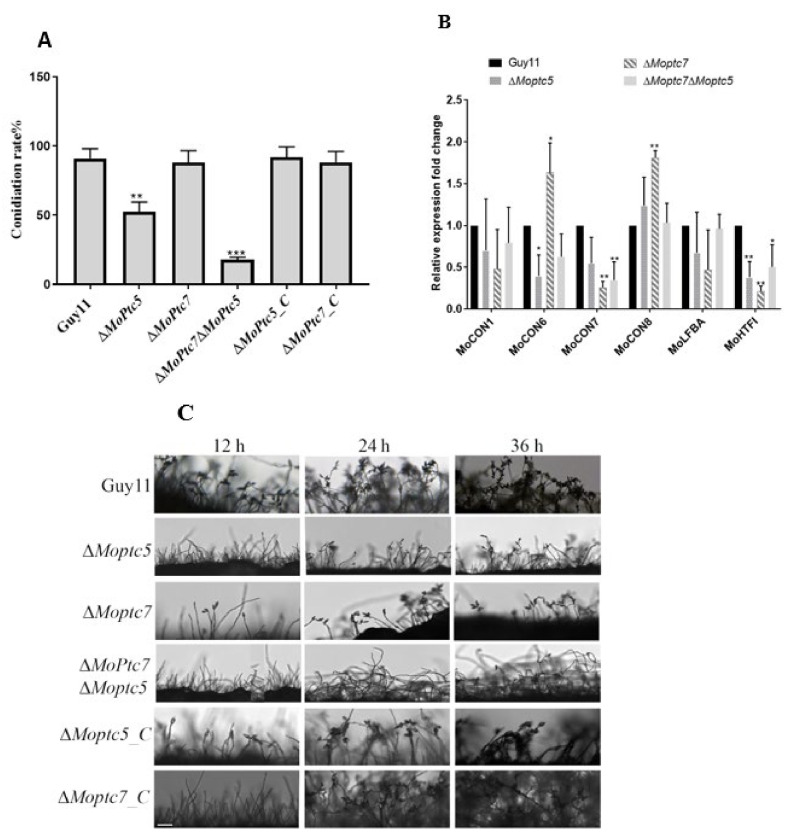
*MoPTC5* deletion causes reduction in conidiation and conidiophores formation of *M. oryzae*. (**A**) Conidia production of the strains grown on RBM for 10 days period. (**B**) Conidiophores formation from Guy11, Δ*Moptc5,* and Δ*Moptc7*Δ*Moptc5* strains on RBM. Δ*Moptc7*Δ*Moptc5* double mutant shows highly reduced conidiophores formation. (**C**) Relative expressions of genes involved in *M. oryzae* conidiation. The various strains were inoculated in liquid CM and incubated at 28 °C under constant shaking at 110 rpm for 3 days, after which total RNAs were extracted for qPCR analysis. Tubulin gene was used as a control. Results are means obtained from three independent replicates. Error bars represent standard deviations while asterisks show significant differences at different *p*-values (*, *p* < 0.1; **, *p* < 0.01; ***, *p* < 0.001)). The data were analyzed by one-way ANOVA using Tukey’s multiple-comparison test.

To understand the roles of MoPtc5 and MoPtc7 in *M. oryzae* conidiation, we cultured the controls and mutant strains on RBM for 10 days period, and monitored conidia development. From our results, we found that Δ*Moptc7* produced almost similar quantity of conidia as the WT. However, conidia production in the Δ*Moptc5* and Δ*Moptc7*Δ*Moptc5* mutants was significantly reduced compared to Guy11 (Figure 4A). Since conidia are always borne on conidiophores, we decided to check for conidiophore formation of the Δ*Moptc5* and Δ*Moptc7*Δ*Moptc5* mutants at 12 h, 24 h and 36 h time points. The results indicated that the number of conidiophores in both Δ*Moptc5* and Δ*Moptc7*Δ*Moptc5* mutants were highly reduced especially at 12 h and 24 h compared to the controls (Figure 4B). We further conducted qPCR to check the expressions of some conidiation-responsive genes, including *MoCON1*, *MoCON6*, *MoCON7*, *MoCON8*, *MoFLBA* and *MoHTFI*. We found that the expressions of *MoCON1*, *MoCON6*, *MoCON7* and *MoHTFI* were downregulated in Δ*Moptc5* and Δ*Moptc7*Δ*Moptc5* mutants (Figure 4C). Put together, we conclude here that MoPtc5 is important for *M. oryzae* asexual reproduction.

### 3.5. MoPtc5 and MoPtc7 Are Involved in Cell Wall Stress Resistance and Dephosphorylation of MoMps1

**Figure 5:**
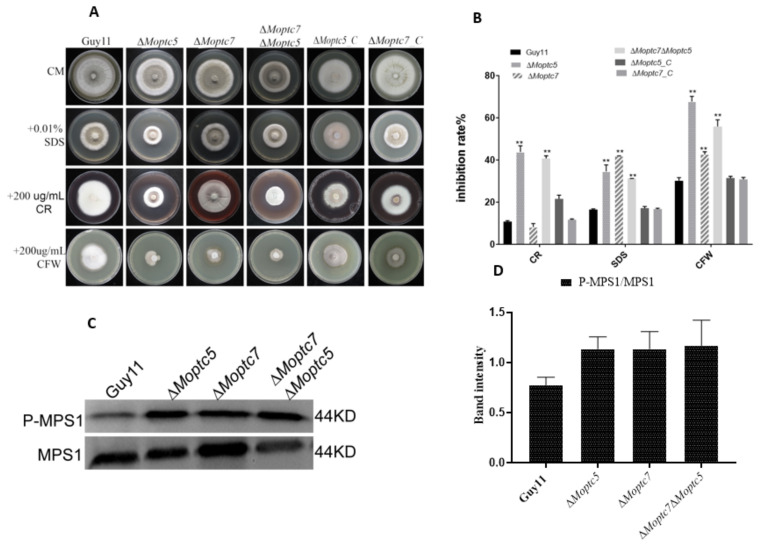
MoPtc5 and MoPtc7 are involved in stress resistance in the blast fungus. (**A**) Colony diameters of the strains were measured 10 days post inoculation on CM media containing cell wall-perturbing agents. (**B**) Growth inhibition rates of the mutants due to the effects of the cell wall stressing substances (Inhibition rate = (the diameter of untreated strain − the diameter of treated strain)/(the diameter of untreated strain) × 100%. Three independent replicates were involved. Error bars represent standard deviations while asterisks show significant differences at different *p*-values (** *p* < 0.01). The data were analyzed by one-way ANOVA using Tukey’s multiple-comparison test. (**C**) MoMps1 phosphorylation level in Δ*Moptc5*, Δ*Moptc7* and Δ*Moptc7*Δ*Moptc5* mutants. (**D**) Comparison of band intensities for MoMps1 phosphorylation levels in the various strains as calculated using imaging software. Despite the obvious increase in the phosphorylation levels, the differences were not significant compared to the wild type.

To analyse the effects of cell wall stress on Δ*Moptc5*, Δ*Moptc7* and Δ*Moptc7*Δ*Moptc5* mutants, we supplemented sodium dodecyl sulphate (SDS, 0.01%), Calcofluor White (CFW, 200 µg/mL) and Congo red (CR, 200 µg/mL) to separate CM growth media. We then cultured the fungal strains on these media and incubated for 10 days in the dark at 28 °C, after which the colony diameters of the cultures were measured. Growth inhibition rates were calculated as earlier stated (Materials and Methods). We found that the growths of Δ*Moptc5*, Δ*Moptc7* and Δ*Moptc7*Δ*Moptc5* were affected towards cell wall stress agents (Figure 5A), however Δ*Moptc5* and Δ*Moptc7*Δ*Moptc5* were highly inhibited on media containing SDS, CR and CFW, while Δ*Moptc7* significantly inhibited only by SDS and CFW (Figure 5B), these results suggesting the importance of MoPtc5 and MoPtc7 in stress resistance. Since MoMps1 phosphorylation level is required for *M. oryzae* cell wall integrity and the two type 2C proteins are protein phosphatases [9], we quantified MoMps1 phosphorylation level in the fungal samples by western blot. We recorded an increase in MoMps1 phosphorylation in Δ*Moptc5*, Δ*Moptc7* and Δ*Moptc7*Δ*Moptc5* mutants, although the differences were not significant when compared to Guy11 (Figure 5C,D). We therefore conclude that MoPtc5 and MoPtc7 are involved in stress resistance and MoMps1 dephosphorylation in the rice blast fungus.

### 3.6. MoPtc5 and MoPtc7 May Mediate Chitin Synthesis to Confer Resistance to Cell Wall Stress in the Blast Fungus

**Figure 6:**
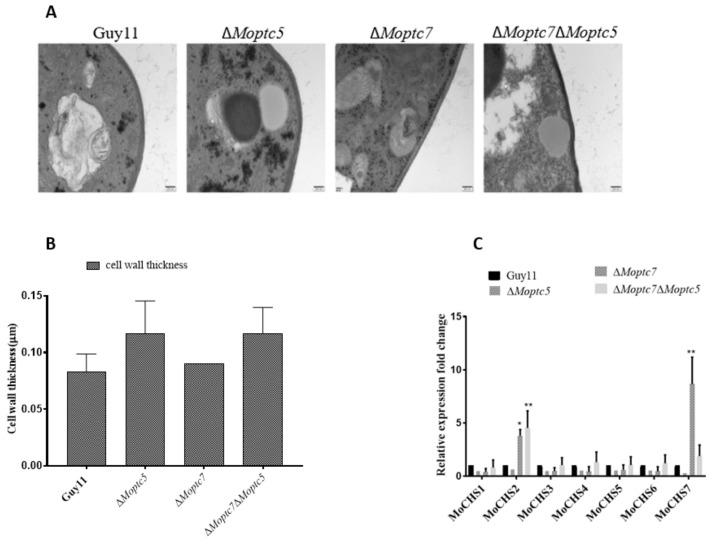
Deletion of *MoPTC5* and *MoPTC7* does not affect cell wall thickness. (**A**) Transverse sections of hyphae from Guy11, Δ*Moptc5*, Δ*Moptc7* and Δ*Moptc5*Δ*Moptc7* strains following transmission electron microscopy. (**B**) Graphs indicating differences in hyphal cell wall thickness between the mutants and Guy11. (**C**) The transcript levels of chitin encoding genes using actin gene as internal control. Three replicates were involved. Error bars represent standard deviations while asterisks show significant differences at different *p*-values (* *p* < 0.1; ** *p* < 0.01). The data were analyzed by one-way ANOVA using Tukey’s multiple-comparison test.

Considering our earlier finding that showed the involvement of MoPtc5 and MoPtc7 cell wall stress resistance, we decided to investigate whether the two proteins contribute to the fungal cell wall thickness. The results from transmission electron microscopy indicated no significant difference in the cell wall thickness of the WT, Δ*Moptc5*, Δ*Moptc7* and Δ*Moptc7*Δ*Moptc5* strains (Figure 6A,B). Since the cell wall integrity is intact, we reasoned that the reduction of mycelial growth on media containing cell wall perturbing agents could have been due to altered chitin synthesis. As such, qPCR assay was performed to check the expression levels of the chitin synthase encoding genes *MoCHS1*, *MoCHS2*, *MoCHS3*, *MoCHS4*, *MoCHS5*, *MoCHS6* and *MoCHS7* in the various strains. We found that the expression of *MoCHS2* was upregulated in Δ*Moptc7* and Δ*Moptc7*Δ*Moptc5*, while *MoCHS7* was highly expressed in Δ*Moptc7*. However, all of these genes were downregulated in Δ*Moptc5* mutant, though with no significant difference (Figure 6C). These results suggest an alteration in chitin content due to *MoPTC5* and *MoPTC7* deletions.

### 3.7. MoPtc5 and MoPtc7 Are Required for Osmotic and Oxidative Stress Tolerance

**Figure 7:**
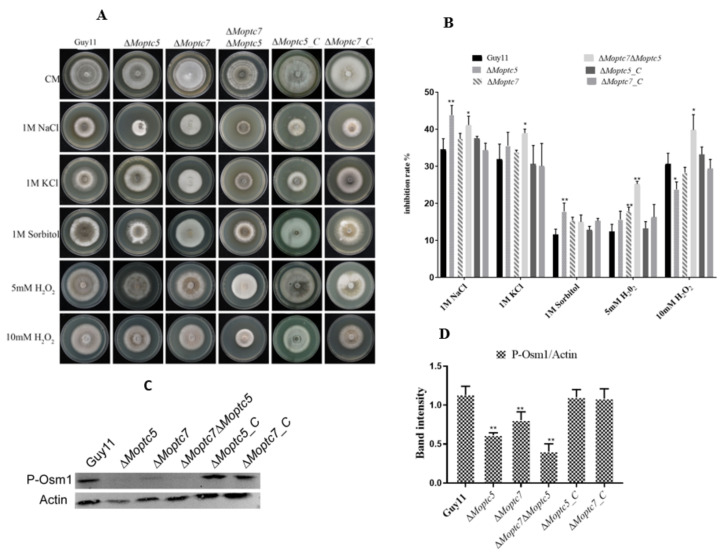
MoPtc5 and Δ*Moptc7*Δ*Moptc5* are indispensable for tolerance to osmotic and oxidative stresses in the blast fungus. (**A**) Colony diameters of the various strains were measured 10 days after inoculation on media containing 1 M NaCl, 1 M KCl, 1 M Sorbitol, 5 mM, 10 mM H_2_O_2_. (**B**) Inhibition rate of the oxidative and osmotic stressors. Inhibition rate = (the diameter of untreated strain − the diameter of treated strain)/(the diameter of untreated strain × 100%). There were three replicates. Error bars represent standard deviations while asterisks show significant differences at different *p*-values (* *p* < 0.1; ** *p* < 0.01). The data were analyzed by one-way ANOVA using Tukey’s multiple-comparison test. (**C**) Osm1 protein abundance in Δ*Moptc5*, Δ*Moptc7*, Δ*Moptc7*Δ*Moptc5* mutants. (**D**) Comparison of band intensities for Osm1/Actin phosphorylation levels in the various strains as calculated using imaging software.

To check the possible involvement of *MoPTC5* and *MoPTC7* in *M. oryzae* tolerance to osmotic and oxidative stresses, we inoculated mycelia plugs from cultures of Guy11, Δ*Moptc5*, Δ*Moptc7*, Δ*Moptc5*Δ*Moptc7* and complementation strains were on media supplemented with 1 M NaCl, 1 M KCl, 1 M Sorbitol and 5 and 10 mM H_2_O_2_ and their colony growths measured. Our results revealed that the growths of Δ*Moptc5* and Δ*Moptc5*Δ*Moptc7* mutants were significantly inhibited by 1 M NaCl while the double mutant Δ*Moptc5*Δ*Moptc7* was highly inhibited by both 5 mM H_2_O_2_ and 10 mM H_2_O_2_ (Figure 7A,B). MoOsm1 is a crucial member of the osmoregulation pathway. A study previously demonstrated that deletion of *MoOSM1* caused hypersensitivity to oxidative stress-inducing agents [30]. To establish its relationship with MoPtc5 and Δ*Moptc7*Δ*Moptc5*, we performed western blot assay of MoOsm1 in Δ*Moptc5*, Δ*Moptc7*, Δ*Moptc7*Δ*Moptc5* and wild type strains. Our results support that the expression of MoOsm1 protein is strongly attenuated in the absence of MoPtc7 and MoPtc5 (Figure 7C,D).

### 3.8. MoPtc5 and MoPtc7 Are Responsible for Appressorium Turgor Generation and Degradation of Glycogen in M. oryzae

**Figure 8:**
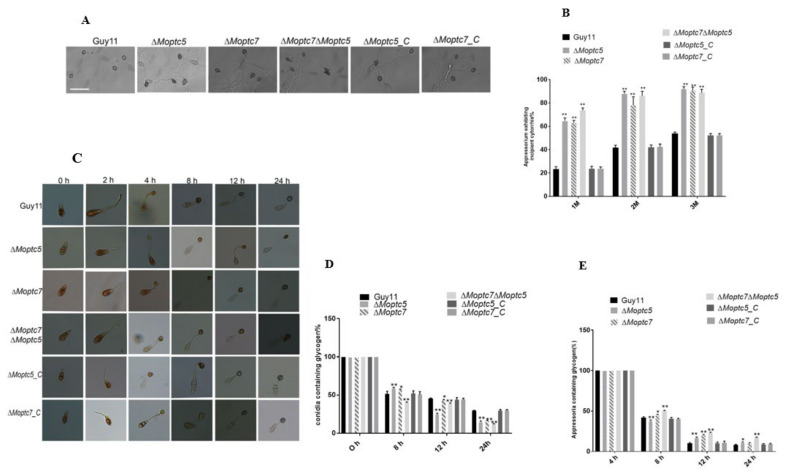
Deletion of *MoPTC5* and *MoPTC7* delayed glycogen mobilization from conidia to appressoria and reduced appressorium turgor pressure. (**A**,**B**) represent the percentages of appressorium exhibiting incipient cytorryhsis after being treated with glycerol. (**C**) Mobilization of glycogen in the mutants and Guy11 at different time points. (**D**,**E**) Micrographs showing percentage of conidia and appressoria containing glycogen.

The involvement of MoPtc5 and MoPtc7 in appressorium-mediated host penetration was also tested by measuring appressorium turgor in Δ*Moptc5*, Δ*Moptc7*, Δ*Moptc5*Δ*Moptc7*, Δ*Moptc5_C*, Δ*Moptc7_C* and Guy11 after induction of appressorium formation on artificial hydrophobic surfaces. They were then incubated for 24 h, after which they were treated with 1 M, 2 M and 3 M glycerol. The number of collapsed appressoria were counted under a light microscope. The results showed that most of the appressoria produced by Δ*Moptc5*, Δ*Moptc7* and Δ*Moptc5*Δ*Moptc7* were collapsed (Figure 8A,B). To examine glycogen degradation, the conidia and appressoria of the strains were stained with KI/I_2_ and glycogen degradation was checked at 2 h, 4 h, 8 h, 12 h and 24 h time points. We found delayed glycogen degradation from conidia to appressoria formation in the mutants (Figure 8C–E).

### 3.9. MoPtc5 and MoPtc7 Are Involved in M. oryzae Pathogenicity

**Figure 9:**
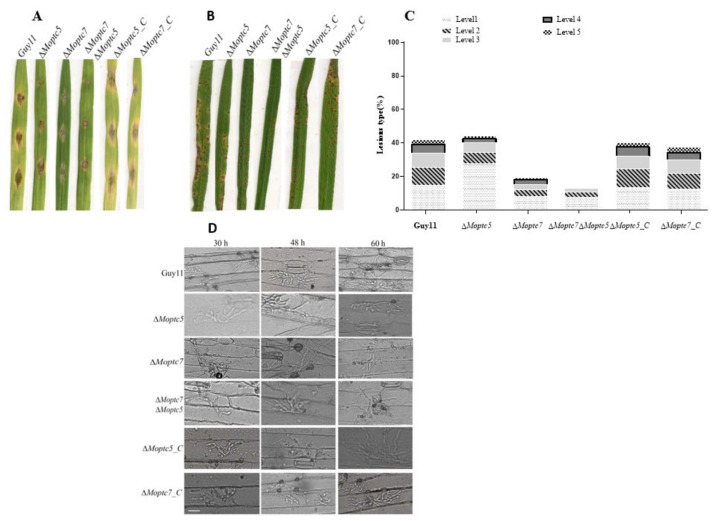
MoPtc5 and MoPtc7 play important roles in *M. oryzae* virulence. (**A**) Pathogenicity assay for Guy11, Δ*Moptc5*, Δ*Moptc7* and Δ*Moptc7*Δ*Moptc5* on barley leaves using drops of conidia suspension. (**B**) The pathogenicity of Δ*Moptc5*, Δ*Moptc7* and Δ*Moptc7*Δ*Moptc5* mutants in comparison to Guy11 using conidia sprays on 3 weeks old barley leaves. (**C**) Extents of lesions development as blast symptoms caused by the indicated strains, Level 1 (uniform dark brown pinpoint lesions without visible centers), Level 2 (small lesions with distinct centers surrounded by a dark brown margin, 1 mm in diameter), Level 3 (small eyespot lesions approximately 2 mm in length with tan centers surrounded by dark brown margins), Level 4 (intermediate size eyespot lesions, approximately 3–4 mm in length), Level 5 (large eyespot lesions approximately 5 mm in length). (**D**) Cuticle penetration defects of the mutants.

We analyzed the virulence of Δ*Moptc5*, Δ*Moptc7*, Δ*Moptc7*Δ*Moptc5*, and Guy11 strains by placing drops of their conidia suspensions on 10 days old barley leaves. At the 7th day of the inoculation, the virulence of the mutants were obviously reduced compared to Guy11 and the complemented strains (Figure 9A). We further harvested spores from these strains to infect 3 weeks old CO39 rice seedlings. The results showed slightly reduced pathogenicity for the mutant strains (Figure 9B,C). To further confirm these results, we investigated the cuticle penetration potential of the mutants on barley leaves at different time points. We found that the Δ*Moptc5*, Δ*Moptc7* and Δ*Moptc7*Δ*Moptc5* mutants had weak cuticle penetrating capacity at 30 h and 48 h time points relative to Guy11 (Figure 9D), which is consistent with their defects in appressoria formation earlier observed. These results reveal the importance of MoPtc5 and MoPtc7 in the pathogenicity of *M. oryzae*.

### 3.10. Subcellular Localization of MoPtc5 and MoPtc7 Genes

**Figure 10:**
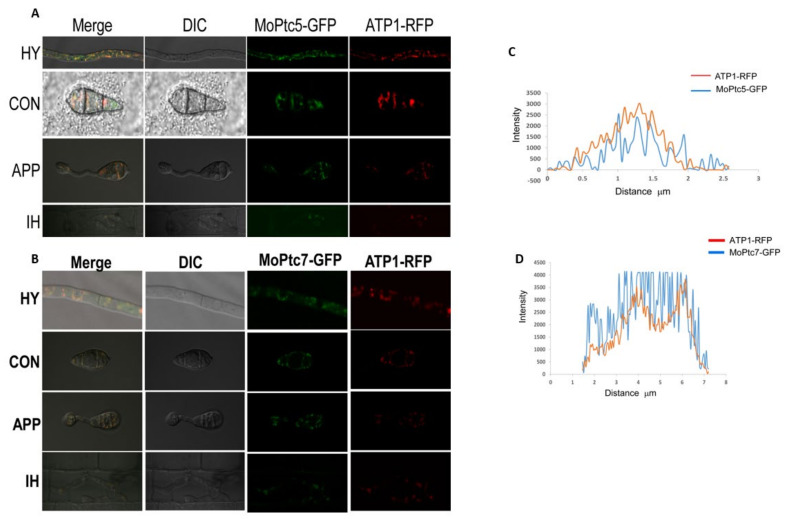
Localization of MoPtc5 and MoPtc7 in rice blast fungus. (**A**,**B**) displays co-localization of MoPtc5, MoPtc7 in hyphae, conidia, appressorium and in plantae as it co-localizes with ATP1_RFP (Red Fluorescence Protein) mitochondria marker. (**C**,**D**) graphs representing intensity fluorescence and distance for MoPtc5 and MoPtc7 co-localization. The pictures were taken by a NikonA1 confocal microscope (Scale bar = 20 µm).

To evaluate the location of MoPtc5 and MoPtc7 in this fungus, we amplified MoPtc5 and MoPtc7 fragments from Guy11 genomic DNA and cloned the two separate fragments in the KpnI/HindIII site of pKNTG plasmid which is fused with GFP. We transformed these constructs separately into the protoplasts of their respective mutants. It was observed that both MoPtc5 and MoPtc7 are localized in mitochondria at all developmental stages. To confirm the mitochondria localization, we cotransformed ATP1 vectors fused with Red fluorescent protein (RFP) and MoPtc5-GFP, MoPtc7-GFP vectors in Guy11 protoplast. The transformants were selected using hygromycin and geneticin antibiotics. We screened the transformants by PCR method and fluorescence microscopy and observed that a colocalization of MoPtc5-GFPand MoPtc7-GFP with the mitochondria marker in hyphae, conidia and in appressorium (Figure 10).

## 4. Discussion

Type 2C protein phosphatases perform various functions in cell biology and signal pathways in eukaryotes. However, their roles in filamentous fungi are poorly known. In this study, we analyzed the functions of MoPtc5 and MoPtc7 to unveil their individual and combined roles in the development and pathogenesis of *Magnaporthe oryzae*. We showed through phylogenetic analysis that MoPtc5 shares high percentage similarity to *S. cerevisiae*, *F. graminearum* and *F. oxysporum* while MoPtc7 is closest to *F. graminearum* and *B. cinerea* (Figure 1B). We also showed the presence of PP2C and PP2C_SIG domains in MoPtc5 and MoPtc7 proteins (Figure 1A). The PP2C and PP2C_SIG domains are conserved in all type 2C protein phosphatases. In this study, we demonstrated that both MoPtc5 and MoPtc7 proteins are localized to mitochondria in *M. oryzae* at different development stages, including hyphae, conidia, appressoria and in infected plants. 

Transcriptomic analyses demonstrated that MoPtc5 and MoPtc7 are differentially expressed at 12 hpi, while at 24 hpi MoPtc7 is up-regulated. These results suggest that MoPtc7 is more active in planta than MoPtc5. Furthermore, we also established that *MoPTC5* and *MoPTC7* genes do not influence *M. oryzae* vegetative growth. However, double deletion of both genes in a single mutant resulted in significantly reduced *M. oryzae* mycelial growth on SYM and RBM. These findings are consistent with that reported by [28]. Which revealed that double deletion of MoPtc5 and MoPtc7 disrupted the growth of *S. cerevisiae*. This simply shows the redundant functions of the two proteins in the vegetative growth of the fungus. 

Conidia formation is very crucial during the lifetime and epidemics of *M. oryzae*, since conidia serve as primary inoculums during host infection [7]. The *M. oryzae* mutant lacking *MoPTC5* gene showed slightly reduced conidiation rate while deletion of *MoPTC7* had no adverse defect on conidiation. However, double knockout of the two genes resulted in a mutant that displayed significant impairment in conidiation rate. These results are consistent with the observed down-regulation of conidiation responsive genes and poor conidiophore developments in the double mutant strain. Therefore, the protein phosphatases might play overlapping roles in asexual reproduction in *M. oryzae*.

Fungal cell wall is a very powerful structure that acts as a barrier that maintains cellular integrity; it is involved in signal transduction, and plays a key function in selective permeability of the cells [31]. In addition, cell wall is important for response to external stresses [32]. In this study, we examined the influence of some cell wall-damaging agents including Calcofluor white (CFW), SDS and Congo red (CR) on Δ*Moptc5*, Δ*Moptc7* and Δ*Moptc7*Δ*Moptc5* mutants. Our results indicate that deletion of *MoPTC5* as well as the double deletion of *MoPTC7* and *MoPTC5* resulted in increased CFW and CR. However, these observations and previous findings on the type 2C protein phosphatases in *S. cerevisiae* [16] revealed the essential function of MoPtc5 and MoPtc7 in cell wall integrity. In addition, double deletion of *MoPTC5* and *MoPTC7* has no effect on the fungal cell wall thickness, but altered the expressions of the chitinase-encoding genes *MoCHS2* and *MoCHS7* which became up-regulated in the double deletion mutant Δ*Moptc7*Δ*Moptc5.* Taken together, these results suggest that MoPtc5 and MoPtc7 synergistically modulate chitin biosynthesis in *M. oryzae*.

Reactive oxygen species (ROS) production and homeostasis are important for general fungal development [33]. In this research work, the double mutant Δ*Moptc7*Δ*Moptc5* was significantly more sensitive to different concentrations of hydrogen peroxide (5 mM H_2_O_2_ and 10 mM H_2_O_2_) than Δ*Moptc7* and Δ*Moptc5* mutants. Based on these results we speculated that MoPtc5 and MoPtc7 might play important roles in the fungal tolerance to oxidative stress. A previous study demonstrated that these type 2C phosphatases are required for osmotic stress tolerance in yeast and in higher eukaryotes [16]. In *M. oryzae*, lacking both proteins showed higher NaCl and KCl sensitivities. Therefore, we reasoned that the type 2C phosphatase gene might redundantly confer tolerance to osmotic stress in *M. oryzae*.

More so, we observed reduction in the fungal pathogenicity due to single as well as double deletions of the type 2C genes in *M. oryzae*. Δ*Moptc7* and Δ*Moptc5* mutants’ virulence in rice and barley was slightly reduced. However, double deletion of *MoPTC7* and *MoPTC5* resulted in highest reduction in *M. oryzae* pathogenicity. We reasoned that MoPtc5 and MoPtc7 might be playing a synergetic role in rice blast disease morphogenesis. Hence, we concluded that MoPtc5 and MoPtc7 may play redundant roles in *M. oryzae* pathogenicity.

Glycerol accumulation in an appressorium generates turgor pressure that is used to physically break the host cuticle for penetration [34]. A previous study indicated that maintenance of turgor pressure is necessary for *M. oryzae* penetration into the host [31]. We therefore tried to investigate whether the observed reduction in the pathogenicity of the mutants was due to reduced turgor generation in their appressoria. Our results showed that Δ*Moptc5*, Δ*Moptc7* and Δ*Moptc7*Δ*Moptc5* mutants had attenuated appressorium turgor pressure. Furthermore, glycogen degradation and transportation from conidia to mature appressoria are key biochemical processes that promote appressorial efficiency and cuticle penetration [35]. We found here that double deletion of *MoPTC7* and *MoPTC5* delayed the degradation and transport of glycogen from conidia to mature appressoria. As such, we speculated that the type 2C phosphatases MoPtc5 and MoPtc7 are positively involved in pathogenesis in *M. oryzae* by regulating glycogen trafficking pathways. 

In summary, the type 2C protein phosphatases MoPtc5 and MoPtc7 in *M. oryzae* play overlapping roles in vegetative growth, conidiation, stress tolerance, pathogenicity, and MoMps1 phosphorylation. The findings unveil additional targets for the control and management of the globally devastating blast disease of rice.

## Data Availability

Not applicable.

## Supplementary Materials

The following supporting information can be downloaded at: https://www.mdpi.com/article/10.3390/jof9010001/s1, Figure S1, Southern blot for confirmation for Δ*Moptc5*, Δ*Moptc7* and Δ*Moptc7*Δ*Moptc5* mutants; Table S1, List of primers used in this project.
