# Supplementary material for: Type 2C Protein Phosphatases MoPtc5 and MoPtc7 Are Crucial for Multiple Stress Tolerance, Conidiogenesis and Pathogenesis of Magnaporthe oryzae
Source: J Fungi (Basel). 2022 Dec 20;9(1):1. doi: 10.3390/jof9010001 (PMC9863299; doi:10.3390/jof9010001)
Supplement: Supplementary file 1 [file jof-09-00001-s001.zip › Table S1.pdf]

PRIMERS USED IN THIS STUDY

List of primers used to generate mutants and complementation

Name	Sequence (5'-3')	Remarks
**MGG_00166AF**<br>**MGG_00166 AR**<br>**MGG_00166 BF**<br>**MGG_00166 BR**	**GCCAAAATGAGATACCAGAC**<br>**AGGGAACAAAAGCTGGGTACCCAGAGACGGTTGCAGAGACAC**<br>**GAATAGAGTAGATGCCGACCGCGGGTTGAGAAACAGCCCGCATAG**<br>**TTCAACGACCACGAAAGC**	Primers used to amplify A and B Fragments. (MGG_00166)
**MGG_00166 OF**<br>**MGG_00166 OR**<br>**MGG_00166 UA**	**AAAACCACAGCCACTCCG**<br>**CGCTTGCTTGTCAAATCG**<br>**CGGTCGGTGGCGGTAGTGAT**	Primers used to check to check ORF (MGG_00166)
**MGG_03154 AF**<br>**MGG_03154 AR**<br>**MGG_03154 BF**<br>**MGG_03154 BR**	**AATCCAATGGTTCCTTCTT**<br>**TTGACCTCCACTAGCTCCAGCCAAGCCCCGACTACATGAATTGACT**<br>**GAATAGAGTAGATGCCGACCGCGGGTTATTCCTTATGTCCAGTAATCC**<br>**GCCTACGATGTTGTTGAG**	Primers used to amplify A and B Fragments (MGG_03154)
**MGG_03154 OF**<br>**MGG_03154 OR**<br>**MGG_03154 UA**	**AGAACGAGCAGTCCTATA**<br>**CCTACTACTTCCTCATTGG**<br>**GCGTGCCCAGTATAGAGC**	Primers to check ORF of (MGG_03154)
**MGG_00166Com-F**<br>**MGG_00166 Com-R**<br>**MGG_03154 Com-F**<br>**MGG_03154 Com-R**	**GAACAAAAGCTGGGTGAGAGGAGGCGCGTTTT**<br>**CTGCAGGCATGCAAGTTGAAGATGTGGCCGGTT**<br>**GAACAAAAGCTGGGTGTCGCAATACTCGGTCTT**<br>**CTGCAGGCATGCAAGGACCTTGATATCCTCG**	Primers used for complementation

A list of quantitative real-time PCR (qRT PCR) primers

Name	Sequence (5'-3')	Remarks
**MoPTC1 QF**<br>**MoPTC1 QR**<br>**MoPTC2 QF**<br>**MoPTC2 QR**<br>**MoPTC5 QF**<br>**MoPTC5 QR**<br>**MoPTC6 QF**<br>**MoPTC6 QR**<br>**MoPTC7 QF**<br>**MoPTC7 QR**<br>**Tubulin 2QF**<br>**Tubulin 2QR**	**CATCCCTATACGACCGAAACTG**<br>**CCGCTGGGTCTTCGATATTT**<br>**CGACAGAGGACCAGACAAATAA**<br>**TCACAGCAGCGTCAATGT**<br>**GATGGAAAGGACAGGTTT**<br>**GTTACGAACGAGATGAGT**<br>**TTCATCAGTTACATCAGCAA**<br>**ACGAAGTAGTTGTGGAAG**<br>**GCCAAGGAAGTCAAGAAA**<br>**TTGTCGTTGCTATTGTCT**<br>**TCGACAGCAATGGAGTTTAC**<br>**AGCACCAGACTGACCGAAGAC**	qPCR primers for PTC genes

**MoCHS1 QF**<br>**MoCHS1 QR**<br>**MoCHS2 QF**<br>**MoCHS2 QR**<br>**MoCHS3 QF**<br>**MoCHS3 QR**<br>**MoCHS4 QF**<br>**MoCHS4 QR**<br>**MoCHS5 QF**<br>**MoCHS5 QR**<br>**MoCHS6 QF**<br>**MoCHS6 QF**<br>**MoCHS7 QR**	**AGTGCGTTCGGATACGTTAG**<br>**TGGTCACCGTGGAAGTATTG**<br>**CGGTCCTCTTAGCCAGTATTTC**<br>**TACGATCCTCAGCCAGATACA**<br>**CAACGAGGACGAGGTTCTTT**<br>**TCTTCTTCCATGCCTCCTTTC**<br>**GAAGAGCTACGCGACAAGAA**<br>**CTCGAAGCATTCAGCGATTTG**<br>**GACTCTTGTTGGCGGTCTTTA**<br>**CATCCCGGATACGAGCTAATTG**<br>**CTTCGCTGGTGAGGTTGAATA**<br>**GCCTACGATGTTGTTGAG**<br>**TTCAACGACCACGAAAGC**	qPCR primers for<br>chitin synthase encoding genes.
**MGG_00184(MoHTFI) QF:**<br>**MGG_00184(MoHTFI)QR:**<br>**MGG_00513(MoCON8) QF:**<br>**MGG_00513(MoCON8) QR**<br>**MGG_05287(MoCON7) QF:**<br>**MGG_05287(MoCON7) QR:**<br>**MGG_02246(MoCON6) QF:**<br>**MGG_02246(MoCON6) QR:**<br>**MGG_01215(MoCOM1) QF:**<br>**MGG_01215(MoCOM1) QR**<br>**MGG_14517(MoflbA) QF:**<br>**MGG_14517(MoflbA) QR:**	**5' AAATGTTATGACGAAAGAG 3'**<br>**5' TTGTGTTGAAAGTTGAAG 3'**<br>**5' ACCTCTTCCTCTTCTTCG 3'**<br>**5' TTGGTCTGTTCGAGTGAT 3'**<br>**5' CGATACGAAGAAATTGAG 3'**<br>**5' GGATCTCCTTAAACTCTT 3'**<br>**5' AAGCAGCACTCGAAGAAGGT 3'**<br>**5' ACATCGCCGCCATCAAAC 3'**<br>**5' AACCAATGCTTCCGATAT 3'**<br>**5' TGAGTCGTTGTAGTATGC 3'**<br>**5' ACAACCTTACAACGATAC 3'**<br>**5' TAACATGCTAACAATTAACG 3'**	qPCR primers for conidiation<br>related encoding genes.
**MGG_03154Probe AF:**<br>**MGG_03154Probe AR:**<br>**MGG_00166 Probe AF**<br>**MGG_00166 Probe AR**	**5'AATCCAATGGTTCCTTCTT 3'**<br>**5'CCGACTACATGAATTGACT 3'**<br>**5'CGGAGTTGGTGTTAATACT 3'**<br>**5'CGATAGGTGACGAAAGATGT 3'**	Primers used to amplify<br>Probe sequences
