# Supplementary figures and images for: Type 2C Protein Phosphatases MoPtc5 and MoPtc7 Are Crucial for Multiple Stress Tolerance, Conidiogenesis and Pathogenesis of Magnaporthe oryzae
Source: J Fungi (Basel). 2022 Dec 20;9(1):1. doi: 10.3390/jof9010001 (PMC9863299; doi:10.3390/jof9010001)

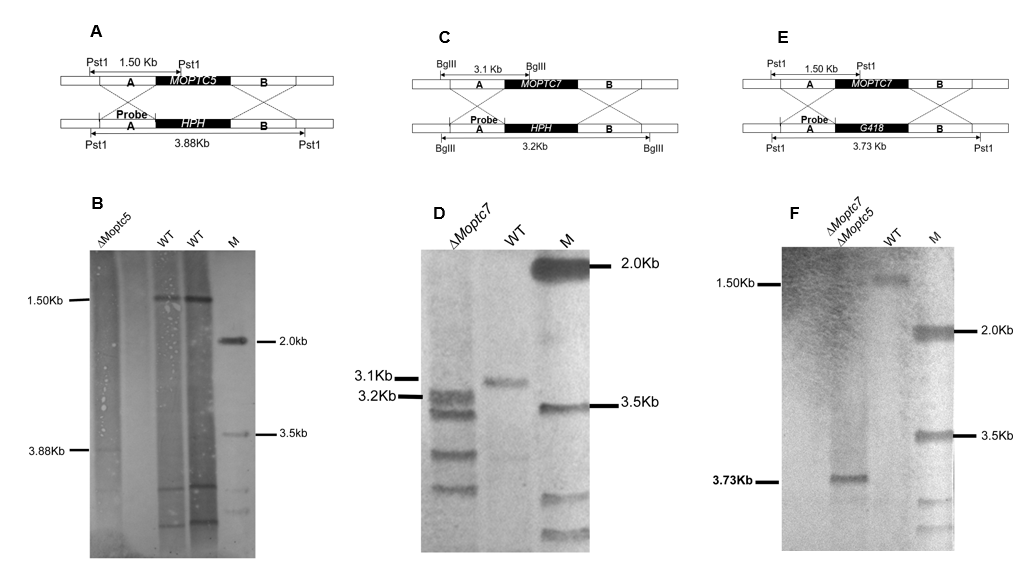

Supplement: Supplementary file 1 [file jof-09-00001-s001.zip › Figure S1.tif]
